# Advances in Regenerated Asphalt Mixtures

**DOI:** 10.3390/ma16072872

**Published:** 2023-04-04

**Authors:** Yuanyuan Li, Tao Bai, Jizhe Zhang, Yangming Gao

**Affiliations:** 1 School of Civil Engineering and Architecture, Wuhan Institute of Technology, Wuhan 430074, China; 2 Qilu School of Transportation, Shandong University, Jinan 250061, China; 3 School of Civil Engineering and Built Environment, Liverpool John Moores University, Byrom Street, Liverpool L3 3AF, UK

This Special Issue is devoted to research on asphalt pavement materials, including asphalt binders, asphalt mixtures and recycled asphalt pavement (RAP). It called for outstanding papers on asphalt pavement recycling materials and new asphalt pavement materials to expand the current understanding of asphalt pavement materials.

Asphalt binder plays very important role in the asphalt mixture. It binds the granular aggregate together and provides the material with strength, meaning that the performance of the asphalt mixture largely depends on the technical performance of the asphalt binder. With respect to the asphalt binder, it is difficult for the performance of conventional petroleum asphalt to meet the requirements of new pavements. Therefore, the modification of the asphalt binder is suggested to improve the performance of the asphalt binder and asphalt mixture [1,2]. With respect to the asphalt mixture, the main efforts are directed toward the strength of the mixture, with the primary aim of reducing the waste of resources, improving the performance of asphalt materials and reducing the economic pressure on the RAP. A short review of the papers in this Special Issue follows.

Current asphalt pavements have large and complex axle loads, and the performance of asphalt pavements deteriorates rapidly with age. However, the performance of asphalt binders can be significantly improved with the use of asphalt modifiers. The asphalt modifier can be a solid waste, such as crumb rubber (CR), or a rock asphalt, such as Bourdon rock asphalt (BRA). Currently, the uncertainty of CR’s swelling mechanism in asphalt limits the application of crumb rubber modified bitumen (CRMB) in the road field. To overcome this limitation, a comprehensive study was carried out on CRMB, swelling rubber in CRMB (SCR) and recycled asphalt after CR action (CRRB) [3]. It was noted that CR can improve the high-temperature performance, ultra-low-temperature cracking resistance, storage stability and elastic recovery of an asphalt binder. The decrease in the relative content of light components in asphalt binder improved the high-temperature performance of CRRB and worsened its low-temperature performance. The swelling reaction led to an increase in the volume of the CR. Additionally, the CR’s microscopic surface became more complex, the small molecule content was significantly higher and the generation of C=C bonds led to an increase in the toughness of the crumb rubber. A Bourdon rock asphalt modified asphalt mixture (BRA-MAM) demonstrates excellent high-temperature performance and moisture damage resistance, reducing the dependence on petroleum asphalt for conventional asphalt pavements. The segregation problem of the BRA in a Bourdon rock asphalt modified asphalt mixture (BRA-MA) has attracted a great deal of scholarly attention. A study of the physical performance and storage stability of BRA-MA by particle size and content of BRA is reported in this Special Issue [4]. Through simulating the separation process of BRA-MA, the viscosity and high-temperature stability of BRA-MA were negatively correlated with the particle size of BRA and positively correlated with its dosage. The degree of separation of the BRA-MA was positively correlated with the particle size, dosage, storage temperature and storage time of the BRA. Less common asphalt modifiers include the mussel-like adhesive L-Dopa methacrylic anhydride (L-DMA), which reacts with asphalt through physical reactions. The main physical reactions of L-DMA are hydrogen bonding, aromatic ring conjugation, the conjugation of benzene rings and the conversion of catechol to quinone and semi-quinone at high temperatures [5]. Among these reactions, the adhesion of modified asphalt was improved mainly through a hydrogen bond and aromatic ring conjugation, and its high-temperature performance was improved mainly through a hydrogen bond, benzene ring conjugation and catechol. L-DMA can improve the rutting coefficient, creep recovery rate and compound modulus of an asphalt binder. When the dosage of L-DMA is higher than 10%, the low-temperature rutting performance of the asphalt can be improved to some extent.

In addition, asphalt aging is an important research problem with respect to asphalt performance. Heat, ultraviolet radiation, moisture and other external factors can have effects on an asphalt binder, leading to its aging. An aged asphalt mixture demonstrates poor performance and a short life. Herein, thermal oxygen aging, ultraviolet aging, and still-water erosion tests were conducted to investigate the aging mechanism of asphalt under various aging conditions such as high temperature, ultraviolet radiation, and an aqueous solution, respectively [6]. High temperature and UV composite conditions had a positive impact on the high-temperature stability of asphalt, but their impact on low-temperature cracking resistance was negative. At the same time, ultraviolet rays had a greater impact on the physical and chemical performance of an asphalt binder than water. The mechanism of the thermal-oxidative aging of asphalt was the increase in saturated hydrocarbons and aromatic ring substances and the rapid generation of polar components. The influence of ultraviolet radiation on the physical and chemical performance of asphalt decreases with the deepening of aging. It is exciting that this provides a research idea for asphalt regeneration. On asphalt, when the aging effects of heat, ultraviolet rays and water occur in the positive direction, the performance of the asphalt can be restored from the reverse direction; that is, to supplement the components lost due to aging, achieve asphalt regeneration and achieve the reuse of the asphalt pavement materials. As steel slag powder (SSP) can be used as a substitute for a natural aggregate in asphalt mixtures, the application of steel slag to asphalt roads can alleviate the incongruous situation of the annual production and utilization of steel slag. Research on SSP as a substitute for natural mineral powder in an asphalt mixture has also been carried out in which the anti-aging performance of steel slag asphalt mortar (SSP-MAM) was the focus of the research. The thermal oxidation and UV aging performance of SSP-AM can be achieved through the use of antioxidants and UV absorbers to prepare active composite modified asphalt mortar (SSP-MAM) [7]. In SSP-MAM, antioxidants capture the free radicals generated by light and thermal oxygen while ultraviolet absorbers convert ultraviolet rays into heat, improving their thermal oxidation and ultraviolet aging performance.

The purpose of designing a high-performance asphalt binder is to improve the performance of asphalt mixture and ultimately apply it to asphalt pavement to improve the performance and service life of the asphalt pavement. The research in this Special Issue indicates that improving the interaction between the asphalt masterbatch and aggregate can extend the durability of pavement, thereby improving its overall performance [8]. The current axle load environment and traffic requirements of automobiles require asphalt mixtures to have early strength, rutting resistance, and self-compacting performance. A new type of cement emulsified bitumen mixture (CEBM) demonstrates excellent rutting resistance, water stability, and dynamic stability which meet the corresponding requirements well [9]. The research on the strength formation mechanism, mechanical performance, and road performance of CEBM showed that the presence of water had a small impact on the adhesion between emulsified asphalt and aggregates and had a positive effect on the processability of the CEBM. In CEBM, the hydration products of cement form a skeleton in the aggregate, jointly establishing a spatial network structure with the original adhesion of the asphalt and ensuring the high strength of the CEBM. To ensure that the Marshall stability of the CEBM met the specifications, the curing time of the CEBM was required to be higher than 6 h, and the recommended dosage of emulsified asphalt and cement in CEBM was determined. The performance of asphalt mixtures is affected by various factors, one of which is aggregate gradation (AG). Through the coarse aggregate vibration compaction test, Marshall compaction test, and wheel tracking test of mixtures with different gradings, the effects of AG on the rutting performance (RP) and volume parameters (VPs) of an asphalt mixture were studied [10]. AG has a high impact on the RP and VPs of asphalt mixtures. To achieve a high bone density of asphalt mixtures, the fine and coarse aggregate boundary sieve (BS) should be 2.36 mm, and there should also be an appropriate BS pass rate. This Special Issue also introduces a phosphogypsum-based filler asphalt mixture, which is formed by coupling phosphogypsum with steel slag powder to form phosphogypsum-based filler (PF). When the proportion of phosphogypsum in the steel slag powder was 23%, a PF asphalt mortar (P-AM) achieved the best performance, and the PF had a positive effect on the high-temperature performance and water stability of the asphalt mixture [11].

The research on asphalt mixture cannot remain only at the laboratory level: on-site research is also necessary. The final on-site construction conditions are the last link that determines the performance of the new asphalt mixture pavement. The uncontrollable temperature problem during the construction of asphalt pavement is one of the causes of asphalt pavement damage. Here is a report on the pavement performance and service life issues caused by temperature segregation during paving with asphalt pavement [12]. Through the use of unmanned aerial vehicle infrared thermal imaging technology to collect the temperature distribution of an asphalt mixture pavement construction site and by simulating on-site pavement conditions in the laboratory, it was concluded that the prediction results for the performance of asphalt mixture pavement obtained using unmanned aerial vehicle infrared thermal imaging technology have a high accuracy, and the relationship between the melt temperature, high-temperature stability and water stability of asphalt mixture was determined.

When the asphalt mixture reaches its service life, its road performance fails. At the same time, it is necessary to recover the asphalt pavement material. The recycling of recycled asphalt pavement materials is proposed to realize the resource utilization of recycled asphalt pavement materials, mostly including recycled asphalt and binder recycled asphalt mixture (RAM). To overcome the limitation of a single light oil regenerator on the performance of recycled asphalt, a composite regenerator made of tung oil, dioctyl phthalate (DOP), C9 petroleum resin and organic montmorillonite (OMMT) was used to regenerate aged asphalt [13]. The optimum ratio of the tung oil composite regenerate is tung oil: DOP: C9 petroleum resin: OMMT = 25:5:2:3. An asphalt regenerate at this ratio can promote the anti-aging performance of recycled asphalt and the dispersion and dissolution of polar substances, and the structure and morphology of aged asphalt can also be restored. During the process of regenerating aged asphalt with a tung oil composite regenerate, the macromolecules in the asphalt are destroyed and transformed into small molecules. The road performance of steel slag recycled asphalt mixture (SSRAM) and basalt recycled asphalt mixture (BRAM) has received attention [14]. Compared to RAM, SSRAM demonstrates better fatigue resistance, skid resistance, high- and low-temperature performance, and water stability, as well as excellent durability in high-temperature and water environments. SSRAM had better rutting resistance, fatigue performance, and low-temperature cracking resistance than BRAM.

Of course, the outstanding research in this Special Issue is not the end point of asphalt mixture research. There are still some limitations in the research process, such as the high-cost preparation of L-DMA, which limits its application in optimizing asphalt performance. In the future, new SSP-MAM anti-aging agent substitutes are needed to reduce the economic pressure caused by the extensive use of ultraviolet absorbers and antioxidants, and the engineering implementation and energy consumption of SSRAM must be studied in the future. These should not become frustrating issues. In the future, after breakthroughs in key issues in the continuous, pioneering research on asphalt mixtures, perhaps road scholars will find pleasure.
